# Disparities in financial hardship between LGBTQ+ and non-LGBTQ+ dyads facing dementia

**DOI:** 10.3389/fpubh.2026.1961786

**Published:** 2026-10-07

**Authors:** Sara Jackson, Moroni Fernandez Cajavilca, Kara Dassel, Jacqueline Telonidis, Nicole Fleming, Jia-Wen Guo, Eli Iacob, Fernando Wilson, Jace Flatt

**Affiliations:** 1 College of Nursing, The University of Utah, Salt Lake City, UT, United States; 2 Department of Economics, University of Utah, Salt Lake City, UT, United States; 3 School of Public Health, University of Nevada, Las Vegas, Las Vegas, NV, United States

**Keywords:** Alzheimer’s disease, cost, dementia, financial hardship, LGBTQ (lesbian gay bisexual trans and queer)

## Abstract

**Introduction:**

Alzheimer’s Disease and Alzheimer’s Disease Related Dementias (AD/ADRD) are among the costliest diseases worldwide, with most expenses borne by persons living with dementia (PLWD) and their care partners. For lesbian, gay, bisexual, transgender, queer, and other sexual and gender minorities (LGBTQ+), who often have lower incomes and less savings at baseline, dementia care costs can be financially devastating. Despite rising healthcare costs, no current measure captures dementia-related financial hardship. This study explored how LGBTQ+ and non-LGBTQ+ care dyads experienced AD/ADRD-related expenses in relation to financial hardship and quality of life.

**Methods:**

As part of a larger study, we conducted two 90-min focus groups: one with non-LGBTQ+ PLWD and care partners (*n* = 8), and one with LGBTQ+ PLWD and care partners (*n* = 5). Participants discussed dementia-related costs and their relevance to financial hardship and quality of life, with questions organized around the three domains of the Financial Hardship Typology: material costs, psychological responses, and coping behaviors. Focus groups were audio recorded, transcribed verbatim, and analyzed using qualitative description.

**Results:**

LGBTQ+ dyads’ concerns appeared distinct from those of non-LGBTQ+ dyads, revealing structural and social barriers contributing to financial hardship. LGBTQ+ dyads described loss of community, reliance on chosen family, unrecognized marriage, legal rights as partners, the need for financial powers of attorney, and uncertainty about future caregiving. Participants also raised concerns about affording placement in facilities with LGBTQ+ anti-discrimination policies.

**Discussion:**

These findings illustrate how structural and social barriers shape financial hardship in the context of dementia. For LGBTQ+ dyads, disparities in financial and social resources contributed to debt, delinquency, bad credit, vehicle repossession, and the need to sell property or relocate. These findings will help inform development of a financial hardship measure capable of capturing dementia’s financial impact across a broad range of PLWD and care partners, while remaining inclusive of LGBTQ+ dyads’ unique experiences. Future research should prioritize sampling understudied populations to broaden perspectives on this issue.

## Introduction

Nearly 13 million Americans provide unpaid care for people with dementia (2). Collectively, these care partners spend 19 billion hours providing care to a person living with Alzheimer’s Disease and Alzheimer’s Disease-Related Dementias (AD/ADRD), time that is valued at more than $446 billion dollars0 (2). AD/ADRD are some of the costliest diseases, with the majority of costs shouldered by persons living with dementia (PLWD) and their care partners due to providing informal care and paying for out-of-pocket expenses such as medication. Financial hardship, defined as a combination of financial stress (subjective negative appraisal regarding expenses), financial strain (objective inability to pay bills) and asset depletion (spending down one’s savings or other wealth) is often experienced by both PLWD and their care partners (3).

For those who identify as lesbian, gay, bisexual, transgender, queer, intersex, asexual, or other LGBTQ+ identities—who at baseline experience lower income and wealth (4), a diagnosis of dementia can be financially devastating. Nearly one-third of LGBTQ+ older adults live at or below 200% of the federal poverty level (5, 6). LGBTQ+ older adults are also more likely to be single or live alone, meaning they may rely more heavily on chosen family—defined as a group of non-biological individuals who “deliberately choose one another to play significant roles in each other’s lives” (7, 8). When LGBTQ+ individuals rely upon chosen family as care partners, or designated individuals who serve as support people and informal caregivers rather than traditional familial support (9), they may also rely upon different financial structures than non-LGBTQ+ persons.

Prior research demonstrates that financial hardship in the context of dementia is associated with worse physical and mental health for both PLWD and their care partners (10, 11). Anxiety and depression are two such mental health conditions that are associated with financial hardship (12). As the population ages and increasing numbers of people are identifying as LGBTQ+ (13), there is a greater risk of dementia-related financial hardship and related negative consequences.

Despite projections that AD/ADRD expenses will continue to grow in future years, little knowledge exists regarding what types of expenses have the greatest negative impact on financial hardship and quality of life. Furthermore, we lack a way to measure financial hardship related to dementia and to track the associated impact of dementia-related expenses over time. In cancer settings, the COmprehensive Score for financial Toxicity (COST) (14) is completed by the person with cancer to help professionals in health and social services recommend potential counter measures such as applying for foundation grants. Developing a standardized measure of financial hardship in dementia could improve our ability to monitor financial burden over time, identify individuals most in need of support, and evaluate interventions aimed at reducing financial hardships, similar to approaches used for persons with cancer.

For those facing dementia, financial responsibilities are often turned over to a primary care partner or to multiple care partners as the PLWD loses cognitive function, suggesting the need for a measure that incorporates the perspectives of all involved parties. The disease of dementia also introduces unique expenses not captured by the COST measure. Thus, we need a way to accurately assess the risk of financial hardship across the disease trajectory for PLWD with a broad range of support networks including multiple care partners. As part of a larger parent study that aimed to develop an inclusive measure of financial hardship for LGBTQ+ and non-LGBTQ+ PLWD and their care partners, this study sought to explore how LGBTQ+ and non-LGBTQ+ care dyads experienced AD/ADRD related expenses in association with their financial hardship and quality of life in order to provide the qualitative groundwork for item generation and domain identification. Differences in the financial experiences of LGBTQ+ and non-LGBTQ+ PLWD and their care partners are underexplored. Ultimately, by capturing a broader range of lived experiences that informs our future measure, we will be better able to assess the risk of dementia-related financial hardship as well as prevent future hardship and its negative consequences.

## Methods

Our study followed best practices for developing and validating scales for health, social, and behavioral research and the International Society for Pharmacoeconomics and Outcomes Research (ISPOR) for Patient Reported Outcome (PRO) instrument development (15, 16). The ISPOR guidelines recommend first determining the context of instrument use and developing the research protocol. We considered our target population to be PLWD and their care partners, and determined that separate focus groups for LGBTQ+ and non-LGBTQ+ dyads would be the most appropriate method of data collection in order to better understand any unique aspects of financial hardship for LGBTQ+ PLWD and care partners. After completing these steps for developing new patient-reported outcome instruments, we moved to “Good practice 3,” which entailed conducting focus groups to elicit concepts from participants. This manuscript reports on these focus groups as well as the next step in instrument development (Good practice 4): analyzing qualitative data.

### Conceptual framework

The Financial Hardship Typology (10) guided our study. Briefly, this conceptual framework has three underlying domains including material costs, emotional responses, and coping behaviors. Originally used in assessing financial toxicity in cancer contexts, this typology suggests that financial hardship is a result of material costs including out of pocket expenses and loss of income/wages, emotional responses related to an increase in dementia care costs, and coping behaviors that PLWD and their care partners adopt to manage their dementia care while experiencing increased household expenses during/after cancer care (10).

### Setting and sample

Participants were eligible for this study if they were 18 years or older, spoke English, had reliable internet access, were able to actively participate in an online focus group discussion, and were able to register in a dyad consisting of one PLWD and one care partner. Both dyad members had to be interested in participating in the study to be eligible. PLWD were defined as persons who self-identified as having been diagnosed with any type of dementia but were in the early stages of the disease, allowing their active participation in discussions. Care partners were defined as a friend or relative who paid for dementia expenses and/or provided care for someone with dementia at least once a month in the prior year. LGBTQ+ PLWD were individuals who self-identified as lesbian, gay, bisexual, transgender, queer, and/or another sexual or gender minority.

Our recruitment strategy leveraged national research registries as well as community organizations. We used two national research registries: ResearchMatch™ and TrialMatch™ (1, 2, 17). ResearchMatch™ is a national health research volunteer research registry supported by the U. S. National Institutes of Health as part of the Clinical Translational Science Award program. TrialMatch™ is a free matching service that connects individuals with AD/ADRD, care contributors, and healthy volunteers to authorized clinical research studies. We also engaged with university-affiliated and community-based recruitment strategies in collaboration with the (1) University of Utah Center on Aging, (2) Utah Chapter of the Alzheimer’s Association, (3) Services and Advocacy for Gay, Lesbian, Bisexual, and Transgender Elders (SAGE), and (4) additional community-engaged recruitment practices such as disseminating flyers at local and national events.

Institutional Review Board (IRB) approval was received from the University of Utah prior to the conduct of any study activities. During the eligibility screening process, interested dyads completed an eligibility screening survey and demographic questionnaire via REDCap software (18, 19). Eligible participants were then contacted, and a Zoom meeting was arranged between the study team and the participants. A copy of the consent and assent documents were emailed to participants prior to this Zoom meeting to allow participants to review the study material at their own pace. During this Zoom meeting, the consent and assent forms were reviewed with each dyad, and a study team member discussed any questions or concerns either participant raised before collecting consent from the care partner and assent from the PLWD. Documentation of consent/assent was waived by IRB due to the minimal risk of the study, so consent and assent were obtained verbally. In preparation for the focus group, each participant was sent an email containing the focus group questions (Table 1) and an initial list of dementia-related costs (see Supplementary Table 1) that would be discussed. The research team developed this initial list of dementia-related costs from COST (14) and through literature reviews. All research participants provided their verbal consent once more, immediately prior to the conduct of the focus groups. Focus groups were led by members of the Community Collaboration and Engagement Team (CCET), a core of the Utah Clinical & Translational Science Institute (CTSI). Focus groups were video recorded via Zoom and uploaded into the study team’s secure Box folder.

**Table 1:** List of focus group questions.

Question number	Questions for focus group participants
1	Reviewing the scale below, were there any words that you did not understand?
2	How do these items relate to persons living with dementia and care contributors with whom you have worked? How do you think these items impact their financial hardship and overall wellbeing?
3	What suggestions do you have for improving the list of material conditions? Are there any items that are not on this list that you think should be?
4	What suggestions do you have for improving the list of emotional responses? Are there other psychological reactions that this list did not cover?
5	What suggestions do you have for improving the list of coping behaviors? Are there additional coping behaviors that are not listed?

### Procedures

We conducted two 90-min focus groups, one with self-identifying non-LGBTQ+ PLWD and their care partners (*n* = 8) and one with LGBTQ+ PLWD and their care partners (*n* = 5). Focus groups were conducted by the University of Utah CCET research staff. Both focus groups were recorded, professionally transcribed, reviewed for accuracy, and deidentified. Transcripts were analyzed using qualitative description, with the three domains of the Financial Hardship Typology—material conditions, psychological responses, and coping behaviors—serving as preliminary *a priori* categories. Qualitative description is often utilized in health science research as a way to stay close to the data and describe phenomenon in everyday language (20). Aligned with methods used in qualitative description, data that did not fall within these three categories were examined to determine if they represented a new category or subcategory (20, 21). Initial coding using material conditions, psychological responses, and coping behaviors was performed by utilizing the FRAME framework to document suggestions by participants (22). FRAME is an expanded framework originally designed to report adaptations to evidence-based interventions and as such, helped track participants’ suggestions for dementia-related expenses to include.

Suggestions related to material conditions were grouped together, as were suggested additions to psychological responses and coping behaviors (see Supplementary Table 3 for an example of how FRAME was utilized to group similar suggestions within each category). One FRAME table was developed for each focus group. NVivo coding conducted by one team member (SJ) and verified by three members (JT, NF, KD) was then compared to each FRAME table to ensure every instance of an item had been appropriately captured and coded. No disagreements on coding labels occurred; additional instances of codes were identified through NVivo coding and agreed upon by the study team. The language utilized in describing each of the three domains of financial hardship was then examined across the two focus group transcripts for similarities and differences.

## Results

### Demographic characteristics

The first focus group consisted of four non-LGBTQ+ PLWD and their four care partners (*N* = 8). The second focus group included two PLWD who self-identified as LGBTQ+ and three care partners to LGBTQ+ PLWD (one care partner was a bereaved care partner) (*N* = 5). PLWD were on average 70.50 (SD = 10.54) years old compared to care partners who were on average 59.1 (SD = 15.94) years of age. The majority of care partners were spouses to PLWD (*n* = 5, 71.4%), while two (28.6%) were adult children to PLWD. The average time since dementia diagnosis was 3.67 (SD = 1.21) years. All care partners (*n* = 7, 100%) had provided direct care to the PLWD while five (71.4%) had provided financial support. See Supplementary Table 2 for all sample demographics.

### Code frequencies

Overall, our qualitative descriptive analysis identified 53 instances of codes related to material costs, 42 codes related to psychological responses, and 42 codes related to coping behaviors. Within material costs, we identified 17 subcodes, with the cost of everyday items, medication costs, and obtaining social security disability income being the most frequent occurrences. Psychological responses contained 11 subcodes, the top two most frequent being future financial security, and feelings of abandonment or loss of community related to financial support. Coping behaviors contained 14 subcodes, with the most frequently identified being seeking resources or programs for financial assistance, planning and budgeting, and changes in lifestyle. See Figure 1 for a coding tree depicting each domain of financial hardship and the subcodes contained within.

**Figure 1:**
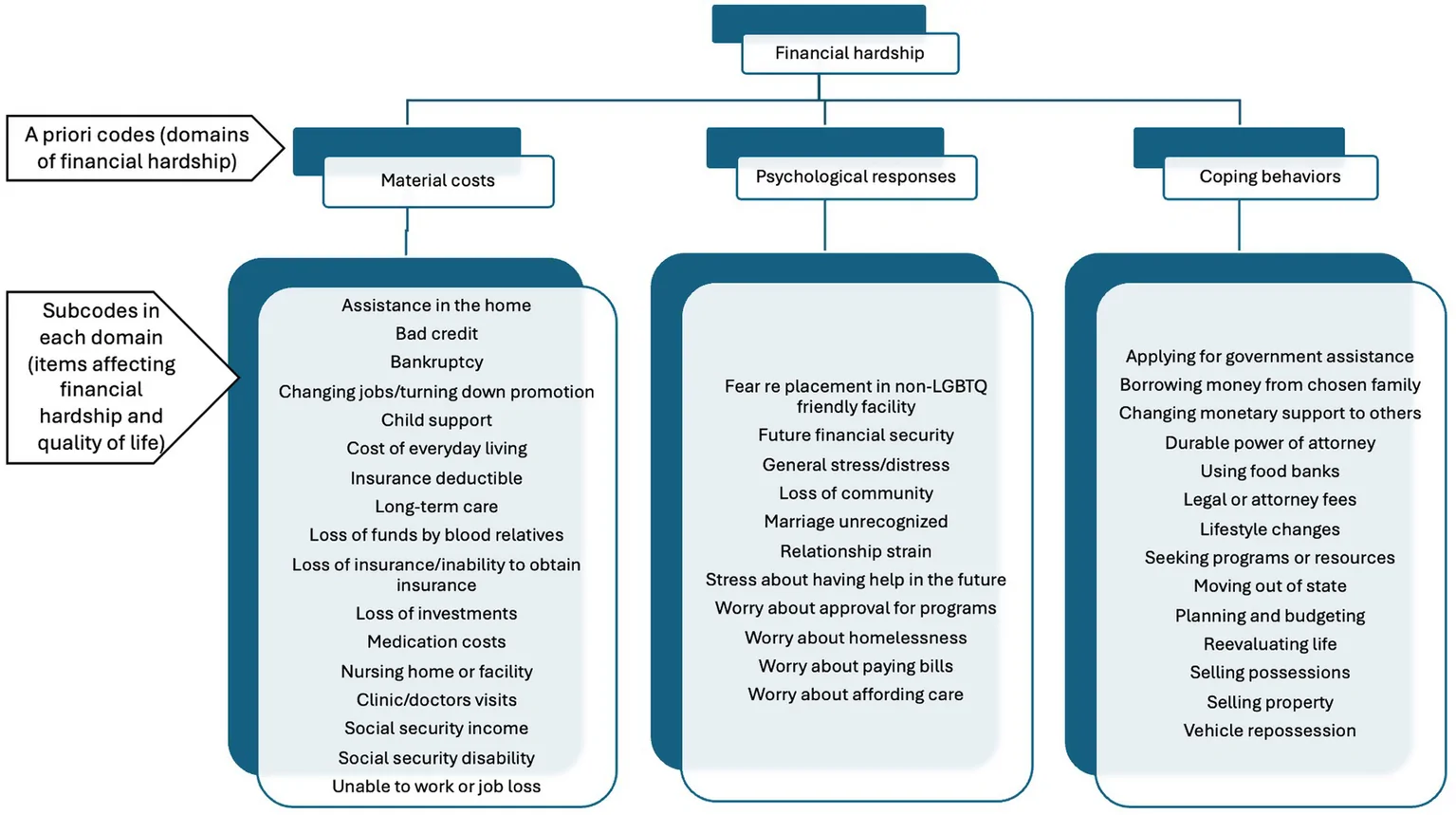
Coding tree illustrating *a priori* codes (domains of financial hardship) and subcodes (items affecting financial hardship and quality of life for PLWD and their care partners).

The data from the LGBTQ+ focus group data contained 24 items that had not been found within the data from the non-LGBTQ+ group. These subcodes included: feelings of abandonment by community, disability income, establishing durable powers of attorney, loss of money due to conflict with blood relatives, unrecognized marriage, applying for government/Veteran Affairs assistance, worry about paying bills, moving out of state, bad credit scores, borrowing money, changing one’s monetary support to others, utilizing food banks, legal or attorney fees, reevaluating what one’s future life would look like given the change in finances, selling possessions, selling property, vehicle repossession, bankruptcy, child support fees, fear about placement in a non-LGBTQ+ friendly long-term care facility, relationship strain, worry about community or government program approval, and worry about homelessness. The non-LGBTQ+ focus group also contained codes not identified in the data of the LGBTQ+ focus group. These subcodes are lifestyle changes, assistance in the home, loss of insurance or inability to obtain insurance, loss of investments, long-term care, and changing jobs/turning down a promotion.

### Qualitative descriptive analysis

#### Similarities

Sentiment across focus groups was similar regarding the relationship between financial hardship and psychological stress, with many dyads describing concerns about how they would pay for future care, especially given the rising costs of healthcare:

We are worried, I mean, worried about how I’m going to [afford] the out-of-pocket expenses. I think the healthcare is super shitty and I do not think it’s going to change with the healthcare prices. How is anybody going to afford healthcare? (LGBTQ+ PLWD, participant ID #111A).

Participants in both focus groups described worrying, stress related to financial troubles, as well as psychological distress related to caregiving and the loss of a job to provide care to the person living with dementia. One participant stated,

I think part of what is missing in this and what [participant name] kind of touched on is, as a caregiver, the onset of caring for someone is unpredictable. In our case our first step was losing a career for one half of the group, and then eventually both of the groups (Care partner of non-LGBTQ+ PLWD, ID # 85B).

Individuals in both focus groups felt that the unpredictable nature of dementia caregiving and dementia-related expenses added to their distress. Participants also described wishing they had planned better for long-term care, they had known how to budget better, and that they had better insurance coverage to pay for a nursing facility, clinic visits, and medical bills.

#### Differences

Differences between LGBTQ+ and non-LGBTQ+ focus groups’ descriptions of financial hardship in the context of dementia were identified in all three domains of financial hardship: material costs, psychological consequences, and coping behaviors.

##### Material costs

One material cost that was specific to the LGBTQ+ focus group was the loss of community due to being shunned or excluded by a church or friend group (due to sexual orientation, dementia diagnosis, or financial circumstances). Similarly, participants in the LGBTQ+ focus group described instances of blood relatives taking over financial support that should have been afforded to them given their relationship but that was taken despite the wishes of the PLWD:

After [name of PLWD] was diagnosed a hundred percent disabled with the VA the family came looking after the money. We did durable power of attorney, which we were advised to do. The family walked in and took her away from me, dumped her in the nursing home, five hours drive away from me…financially that hardship meant that when they took her away, I only had my small pension coming in because they took control of all of her money (Care partner of LGBTQ+ PLWD, ID#182B).

Other participants described their fears that they were not legally married and what this would mean for their future care, insurance benefits and financial circumstances when one dyad member dies. One participant described the financial toll that their previous marriage was still taking on them, as they continue to pay child support. Many participants also described taking initiative in meeting with attorneys in order to put finances in order and ensure that the other dyad member has the legal authority to be the durable power of attorney for the other:

We had to scrounge for that money, but we went and talked to a lawyer, we absolutely did, and to make sure that [name of care partner] was taken care of and that we had a plan, the kids knew what the plan was and that we have it all in legal form. But that is not cheap (LGBTQ+ PLWD, ID#111A).

Dyads in the LGBTQ+ focus group also described experiences of debt, delinquency, and bad credit resulting from dementia-related financial hardship.

##### Psychological responses

The psychological responses resulting from these material costs included feeling insecure due to the prior experience of a partner’s blood family stealing money and not recognizing their relationship, as well as fear that a partner’s children would not help financially or with direct care in the future if the partner was to die first:

[Name of PLWD] and I never technically got married even though we have been together for 21 years and so even though we have power of attorneys and that kind of stuff set up, there’s still that fear of what happens, what happens when he goes or I go, I trust that the kids or somebody’s going to step up and come and take care of us or something. I cannot say that I’m confident that if something happened to [name of PLWD], I would think they’d come take care of me. But if something happened to me, I cannot guarantee that even though he’s been part of their lives all these years (Care partner of LGBTQ+ PLWD, ID#50B).

Participants described concerns about how other LGBTQ+ family members might pay for someone to take care of them given their lack of biological children. Participants also stated that they feared losing their rights or rights not being upheld due to being gay:

If it were not for [name of care partner], I really do not have anyone to take care of me, and I cannot depend on his kids necessarily. Yeah. So I would say because I’m gay and I’ve never been married, if I did not have [name of care partner], I would be lost (LGBTQ+ PLWD, ID#50A).

Similarly, the care partner to this LGBTQ+ PLWD relayed,

In the gay community, it’s like what rights are afforded to us just because we are gay? There’s no guarantees in a marriage situation. Somehow, we need to have rights for each other, even if we are not technically on paper married. Because even though you have everything set up, there’s still that chance that things could all fall apart and you do not have the rights, (Care partner of LGBTQ+ PLWD, ID# 50B).

There was also a concern that if a nursing home was needed, an affordable option would not have an anti-discrimination policy towards LGBTQ+ individuals, and that any facility that was LGBTQ+ friendly would be outside their financial means:

There’s also that level of fear of placing if it’s necessary to place your loved one in a care facility, just because of the fact of our community, there’s a heightened threat of abuse, a heightened threat of neglect, a heightened threat of lack of care. Just because, I mean just because fill in the blank, but just because I think that is a huge, huge scary risk for us (Care partner to LGBTQ+ PLWD, ID#182B).

##### Coping skills

In response to the material costs incurred or expected, as well as the psychological responses experienced because of these costs, participants in the LGBTQ+ focus group detailed unique coping behaviors not described by the non-LGBTQ+ participants. They described accepting financial help from chosen family despite the hesitancy to do so, and legalizing documents establishing financial powers of attorney and other spousal benefits. Unique coping behaviors included the repossession of a vehicle, selling land and property, and moving to another state to be closer to chosen family who may provide care in the future. One participant reported,

The delinquency, she lost [her] car…We had to move out of state,” (LGBTQ+ PLWD, ID#111B) while another recounted, “We just moved back from [name of state]. We just got back because chosen daughter’s birth mom needs help as well, and she could not take care of both of us from a thousand miles apart (care partner to LGBTQ+ PLWD, ID#182B).

## Discussion

The results from the qualitative descriptive analysis of focus group data highlight the differences in how dementia-related costs affected financial hardship and quality of life among LGBTQ+ versus non-LGBTQ+ dyads in our sample. Our findings also shed light on which dementia-related costs most greatly affect financial hardship and quality of life of both the care partners and PLWD. This in-depth information will be used to develop a measure of financial hardship for use by a diverse group of PLWD and care partners facing dementia, including non-LGBTQ+ dyads. Our results are limited by a small sample size of individuals who are primarily Caucasian, have medical insurance, and cohabitate, and therefore findings may not be generalizable to other groups. Only one focus group was conducted with each population, which means that concepts that arose in each group may not necessarily arise in a new sample of dyads. Additionally, given the need for focus group participants to be able to actively participate, findings may not apply for those who are further along in the dementia disease trajectory. Care partners to PLWD with later stage disease were not included in the focus groups, as the inclusion of the entire care network including the PLWD and all care partners was considered central for concept elicitation. Care partners to PLWD in the later stages of the disease will be included in the pre-test and psychometric validation of the measure. (Of note, in the non-LGBTQ+ focus group, one PLWD did not actively participate in the discussion, yet we retained their demographic and health data as they submitted REDCap surveys.)

Our findings may align with prior work demonstrating that LGBTQ+ populations face more structural and social barriers, which shape their financial hardship. For example, participants in the LGBTQ+ focus group described painful experiences of ex-communication from churches, abandonment by friends or biological family, or being shunned by religious or community groups who they had previously relied upon for financial, instrumental, and emotional support. In addition, discrimination in the workplace and legal recognition of partnerships significantly impacted LGBTQ+ participants’ financial and psychological wellbeing as well as their experience that safety nets do not exists for them.

LGBTQ+ dyads in our sample were less concerned about their future financial security as they were more worried about paying for food, utilities, and housing due to the out-of-pocket costs of dementia care. This aligns with prior research findings that four out of five LGBTQ+ care partners reported seeking resources for financial assistance for PLWD (23). These dyads may have been more likely to discuss the receipt of disability income, which could align with prior research demonstrating a greater burden of comorbid disease among this population (24, 25). These findings may also align with demographic data showing that LGBTQ+ groups have lower incomes, fewer opportunities to accrue savings, and lower access to resources (and therefore must rely upon disability income to make ends meet) (4, 26).

For non-LGBTQ+ dyads facing dementia, future financial stability and lifestyle changes were frequently discussed. These items relate to the ability to continue living the life originally planned for and maintaining one’s financial situation in the present and the future. For LGBTQ+ dyads, the inability to work due to dementia or dementia caregiving, obtaining Social Security disability, the loss of community-related financial support, and distress related to their financial situation, illustrates that LGBTQ+ PLWD and care partners in this focus group were less focused on their future financial security and maintaining wealth than they were on their day-to-day expenses and ability to earn income. These differences in present versus future concerns reflect the lack of LGBTQ+ friendly safety nets for financial, instrumental, respite care, and social support needs that LGBTQ+ people feel safe to rely upon in times of need. These results align with other research illustrating that LGBTQ+ persons are less likely to have a spouse or biological children on which to rely (27), are less likely to have accrued savings (28), and are less likely to access formal support services (29). Taken together when LGBTQ+ PLWD must rely upon chosen family for financial support, they often face higher out-of-pocket costs for care than non-LGBTQ+ PLWD who may have biological family and greater access to formal support services (30).

Our results add to the knowledge of economic, social and health disparities experienced by LGBTQ+ persons, as they demonstrate how structural barriers can interact in ways that further exacerbate financial hardship of this population. For example, a lack of biological children, legally recognized spouse or partner, or legally recognized chosen family combined with the lack of financial support from a church or community group and lower access to formal community or government resources can create an exponentially more difficult situation for an LGBTQ+ dyad who is unable to work. These compounding disparities as well as LGBTQ+ specific needs such as the need to ensure that one has legal authority as a durable power of attorney, or the loss of funds due to decreased inheritance, blood relatives not honoring the relationship or recognizing the same-sex marriage, work together as barriers to physical, psychological, and financial wellness.

## Conclusion

While this study was limited by its cross-sectional design and small sample size, limiting the generalizability of findings, the in-depth description of the intersection between material conditions, psychological responses and coping behaviors experienced by LGBTQ+ and non-LGBTQ+ PLWD and their care partners adds new understanding to the field of dementia caregiving and dementia-related financial hardship. Our findings suggest that the inclusion of diverse PLWD and their care partners is crucial in understanding and measuring their unique experiences of dementia-related financial hardship. The results of this study not only demonstrate the need to consider the lived experiences of PLWD and their care partners but also illustrate the critical need for a more robust safety net, particularly for LGBTQ+ PLWD and their care partners given overlapping disparities and barriers to financial and social support. Future studies should consider LGBTQ+ populations as more at-risk of financial hardship and its negative consequences and prioritize tailored interventions for this group. By better understanding the dementia-related expenses that have the greatest impact on quality of life and financial hardship, we aim to create a more inclusive and effective instrument for measuring dementia financial hardship. Ultimately, we plan to use this measure in the identification of families at greatest risk of financial hardship and to prioritize timely, population-specific interventions.

## Data Availability

The datasets presented in this article are not readily available because Data are potentially identifiable given the small sample size and therefore are not available to the public. Requests to access the datasets should be directed to sara.jackson@nurs.utah.edu.
