# Supplementary material for: Disparities in financial hardship between LGBTQ+ and non-LGBTQ+ dyads facing dementia
Source: Front Public Health. 2026 Oct 7;14:1961786. doi: 10.3389/fpubh.2026.1961786 (PMC13684908; doi:10.3389/fpubh.2026.1961786)
Supplement: Supplementary file 1 [file Supplementary_file_1.docx]

**Supplementary Material**

Table 1. Initial dementia-related costs and Likert scale for rating item importance to financial hardship and quality of life

| **#** | **Item** | **Definitions/Examples** | **Rating** |
| --- | --- | --- | --- |
|  | *Material Costs* |  | 0= Not important  1= Mildly important  2= Important  3= Extremely important |
| 1 | Medication | Refers to the cost of paying for any medications | **0 1 2 3** |
| 2 | Inpatient/outpatient visits | The cost of paying for inpatient hospitalizations or outpatient office visits to medical providers | **0 1 2 3** |
| 3 | Insurance co-payments or deductibles | A co-payment is amount one pays upfront when they visit a medical provider  A deductible is the amount insurance requires one to pay before they pay for a covered medical expense | **0 1 2 3** |
| 4 | Transportation to clinic/hospital | Some common examples include bus fare, the cost of gasoline, airfare, Lyft or Uber rides, and similar expenses | **0 1 2 3** |
| 5 | Home-based long-term care | The care one might receive in their home including assistance with daily living activities, personal care, medical services such as wound care or medication management | **0 1 2 3** |
| 6 | Nursing home expenditures | The cost of paying for room and boarding and the care received in a nursing home environment | **0 1 2 3** |
| 7 | Adult day care | The cost of a daytime program that offers activities and supervision for older adults—usually at a facility | **0 1 2 3** |
| 8 | Paid help | The cost of hiring someone to assist with caregiving, respite, cleaning, cooking, etc. | **0 1 2 3** |
| 9 | Reduced/lost income by the patient or the care contributor | Losing income because one can’t work due to dementia, need to care for someone with dementia, or turning down or missing out on a promotion | **0 1 2 3** |
| 10 | Change in productivity or performance at work by patient or care contributor | Decrease in productivity at work or receiving a warning about performance/attendance | **0 1 2 3** |
| 11 | Absent from work | Taking sick leave/vacation days, leaves of absence, FMLA, unpaid leave, arriving late to work, leaving work early, receiving a warning about work attendance | **0 1 2 3** |
| 12 | Change in employment status | Early retirement/retired early, went from full to part-time, cut back hours, gave up work entirely, or the loss of a job | **0 1 2 3** |
| 13 | Out of pocket expenses | Any type of item one may need to purchase with their own money  Also includes services that are not covered by insurance | **0 1 2 3** |
| 14 | Medical debt/bankruptcy | Medical debt means unpaid medical bills  Bankruptcy happens when one can’t pay these bills or are so late in paying them that they are sent to a collection agency | **0 1 2 3** |
| 15 | Using savings to pay for medical care, borrowing from friends/family members, taking out loans | Paying for any type of medical care by using money from one’s personal savings or borrowing money from others | **0 1 2 3** |
| 16 | Home modifications or durable medical equipment | Home modifications, like adding handrails or ramps  Durable medical equipment includes items like wheelchairs, walkers, and oxygen tanks | **0 1 2 3** |
| 17 | Sold possessions | Selling one’s belongings in order to pay bills and get by financially | **0 1 2 3** |
| 18 | Susceptibility to fraud/scams | Having trouble spotting and avoiding scammers who want to steal one’s money | **0 1 2 3** |
| 19 | Payment delinquency or default | Payment delinquency means missing one or more payments for debt such as a home or auto loan  Default means breaking the terms of a loan by not paying what one agreed to pay | **0 1 2 3** |
| 20 | Low credit scores | Having a credit score that is too low to get the best or “prime” interest rates; this can cause more difficulty securing loans | **0 1 2 3** |
|  | *Emotional Responses* |  |  |
| 21 | Worsening quality of life for the patient or care contributor | Quality of life refers to the degree to which a person is healthy, comfortable, and able to participate in and enjoy life events | **0 1 2 3** |
| 22 | Stress, distress, worry about affording care or paying medical bills | Internal anxiety-based feelings related to one’s financial ability to afford care or pay medical bills | **0 1 2 3** |
| 23 | Worrying if one’s income is enough to cover the costs of dementia care | Internal feelings of worry or distress about the ability of one’s earnings to cover dementia-related care | **0 1 2 3** |
| 24 | Distress about future financial security | Internal feelings of anxiety related to one’s future finances and ability to afford care | **0 1 2 3** |
| 25 | Stress, distress, worry about who will manage accounts or finances | Internal feelings of anxiety related to finding and or deciding who will manage money and or trusting someone else to manage money | **0 1 2 3** |
|  | *Coping Behaviors* |  |  |
| 26 | Delaying/forgoing medical care | Delaying or skipping medical care because of worries about the cost and being able to afford it | **0 1 2 3** |
| 27 | Not taking medications as prescribed | Skipping doses, taking less medication than prescribed,  avoiding filling prescriptions due to concerns regarding cost of medicine | **0 1 2 3** |
| 28 | Changing how you spend money | Changing how one usually spends money to manage finances and the extra cost of dementia care. This does not mean making unusual or risky financial decisions because of changes in their thinking | **0 1 2 3** |

Table 2. Demographic characteristics of persons living with dementia (PLWD) and care partners (CPs)

| **Care partner (CP) & Person living with dementia (PLWD) characteristics** | | | | |
| --- | --- | --- | --- | --- |
|  |  | **PLWD**  n= 6 | **CP**  n= 7 |  |
|  |  | Mean (SD) | Mean (SD) |  |
|  | Age in years | 70.50 (10.54) | 59.14 (15.94) |  |
|  | Diagnosis Duration in years | 3.67 (1.21) | -- |  |
|  | Household Size | 2.17 (0.98) | 2.33 (0.82) |  |
|  |  | n (%) | n (%) |  |
|  | **PLWD Dementia Diagnosis** |  |  |  |
|  | Alzheimer’s disease | 3 (50.0) | -- |  |
|  | Vascular dementia | 1 (16.7) | -- |  |
|  | Other: | 2 (33.3) | -- |  |
|  | **PLWD Direct Care** |  |  |  |
|  | Once every few months | 1 (16.7) | -- |  |
|  | Every week | 1 (16.7) | -- |  |
|  | Every day | 4 (66.7) | -- |  |
|  | **CP relation to PLWD** |  |  |  |
|  | Parent | -- | 0 |  |
|  | Child | -- | 2 (28.6) |  |
|  | Spouse or long-term partner | -- | 5 (71.4) |  |
|  | **CP gives Direct Care** |  |  |  |
|  | Yes | -- | 7 (100.0) |  |
|  | **CP gives financial Support** |  |  |  |
|  | Yes | -- | 5 (71.4) |  |
|  | No, I have not provided financial care | -- | 2 (28.6) |  |
| Race | | | | |
|  | White/Caucasian/European Descent | 6 (100.0) | 6 (85.7) |  |
|  | Multiracial | 0 (0.0) | 1 (14.3) |  |
| Hispanic | | | | |
|  | No | 6 (100.0) | 7 (100.0) |  |
|  | Yes | 0 (0.0) | 0 (0.0) |  |
| Gender | | | | |
|  | Man | 3 (50.0) | 1 (14.3) |  |
|  | Woman | 3 (50.0) | 6 (85.7) |  |
| Sexual Orientation | | | | |
|  | Straight | 4 (66.7) | 4 (57.1) |  |
|  | Gay | 1 (16.7) | 1 (14.3) |  |
|  | Lesbian | 1 (16.7) | 2 (28.6) |  |
| Education | | | | |
|  | High school graduate (Grade 12 with diploma or GED certificate) | 1 (16.7) | 1 (14.3) |  |
|  | Some college, associate degree, and or vocational/technical school | 1 (16.7) | 3 (42.9) |  |
|  | College or university degree (e.g. BA, BS, AB) | 0 (0.0) | 2 (28.6) |  |
|  | Postgraduate or professional degree (e.g. MA, MS, PhD, MD, JD) | 4 (66.7) | 1 (14.3) |  |
| Marital Status | | | | |
|  | Married/legal civil union | 3 (50.0) | 3 (42.9) |  |
|  | Living with a partner | 1 (16.7) | 1 (14.3) |  |
|  | Divorced | 1 (16.7) | 0 (0.0) |  |
|  | Widowed | 1 (16.7) | 1 (14.3) |  |
|  | Never been married | 0 (0.0) | 2 (28.6) |  |
| Living Situation | | | | |
|  | Live by myself | 1 (16.7) | -- |  |
|  | Live with significant other | 2 (33.3) | -- |  |
|  | Live with significant other and other individuals | 1 (16.7) | -- |  |
|  | Live with a roommate | 1 (16.7) | -- |  |
|  | Other | 1 (16.7) | -- |  |
| Live with PLWD | | | | |
|  | Yes | -- | 6 (85.7) |  |
|  | No | -- | 1 (14.3) |  |
| Employment | | | | |
|  | Part-time | 0 (0.0) | 1 (14.3) |  |
|  | Not employed | 1 (16.7) | 2 (28.6) |  |
|  | Retired | 5 (83.3) | 1 (14.3) |  |
|  | Disability | 0 (0.0) | 2 (28.6) |  |
|  | full-time caregiver | 0 (0.0) | 1 (14.3) |  |
| Household income | | | | |
|  | Less than or equal to $24,999 | 1 (16.7) | 1 (16.7) |  |
|  | $25,000 to $34,999 | 2 (33.3) | 1 (16.7) |  |
|  | $35,000 to $49,999 | 1 (16.7) | 1 (16.7) |  |
|  | $50,000 to $74,999 | 0 (0.0) | 1 (16.7) |  |
|  | $75,000 or higher | 2 (33.3) | 2 (33.3) |  |
| Insurance | | | | |
|  | Individual health insurance (through ACA) | 1 (16.7) | -- |  |
|  | Medicare | 3 (50.0) | -- |  |
|  | Medicaid | 1 (16.7) | -- |  |
|  | Other (VA Insurance and Medicare) | 1 (16.7) | -- |  |

Table 3. FRAME table for tracking participant suggestions

| *Location:*  Material Costs—Change in employment status  *Description of adaptation or modification*:  “Was planning on working until I was 70.”  “And with the Parkinson's, I was just having a real difficult time and I just had to to go.”  “We had the change in employment status.”  Frequency Count: 2 | *Planned:* Was the intervention planned? NA  *Who:* Who made the decision to modify? SGM focus group  *Level:* At what level of delivery was the modification made?  Item development  *Nature:* What is the nature of the modification? Changes in employment for both partners  *Fidelity:* What is the relationship between modification and fidelity? NA | *Goal:* Reasons for making the adaptation  Ensure item captures the experiences of both partners  *Reason:* Sociopolitical, Organization / Setting, Provider, and / or Recipient; can expand further  Participant reported outcomes relevant to both members of the dyad |
| --- | --- | --- |
| *Location*: Material Costs—Medications  *Description of adaptation or modification*:  “The out-of pocket costs and the prescriptions.”  “The timing of medications and different things that there has to be a set schedule.”  “Food to eat so that the medications work.”  Frequency Count: 1 | *Planned:* Was the intervention planned? NA  *Who:* Who made the decision to modify? SGM Focus Group  *Level:* At what level of delivery was the modification made?  Item development  *Nature:* What is the nature of the modification?  Medication costs include prescriptions and over the counter drugs; may also want to include costs of food  *Fidelity:* What is the relationship between modification and fidelity? NA | *Goal:* Reasons for making the adaptation  Revise wording to better reflect participant experiences; add food costs to out-of-pocket expenses  *Reason:* Sociopolitical, Organization / Setting, Provider, and / or Recipient; can expand further  Participant reported outcomes relevant to both members of the dyad |
